# Effect of different stunning methods on antioxidant status, myofibrillar protein oxidation, and gelation properties of large yellow croaker during postmortem

**DOI:** 10.1016/j.fochx.2023.100709

**Published:** 2023-05-10

**Authors:** Yixuan Dong, Hongzhi Zhang, Jun Mei, Jing Xie

**Affiliations:** a College of Food Science and Technology, Shanghai Ocean University, Shanghai 201306, China; b National Experimental Teaching Demonstration Center for Food Science and Engineering Shanghai Ocean University, Shanghai 201306, China; c Shanghai Engineering Research Center of Aquatic Product Processing and Preservation, Shanghai 201306, China; d Shanghai Professional Technology Service Platform on Cold Chain Equipment Performance and Energy Saving Evaluation, Shanghai 201306, China

**Keywords:** Fish slaughter, Stunning stress, Antioxidant status, Myofibrillar protein, Gel properties

## Abstract

Post-mortem muscle biochemical processes play a crucial role on fish fillets quality and they are strictly linked to stunning methods. The improper stunning methods before slaughter could cause the fish to deteriorate more quickly during cold storage. This study aimed to investigate the effect of stunning methods (hit on the head, T_1_; gill cut, T_2_; immersion in ice/water slurry, T_3_; CO_2_ narcosis, T_4_; 40% CO_2_ + 30 % N_2_ + 30% O_2_, T_5_) on myofibrillar proteins (MPs) of large yellow croaker. The results indicated that T_2_ and T_3_ samples were significantly damaged compared with other samples, which reflected that the activities of total superoxide dismutase (SOD), catalase (CAT), and glutathione peroxidase (GPx) were significantly damaged during cold storage in T_2_ and T_3_ samples. And the gill cut and immersion in ice/water slurry resulted in the generation of protein carbonyl, the decrease of Ca^2+^-ATPase, free ammonia and protein solubility, and the production of dityrosine during storage. In addition, MPs gel of T_2_ and T_3_ samples showed the decrease of water hold capacity (WHC) and whiteness, structure destruction, and water migration. The T_4_ samples had the least damage of MPs and gel structure during cold storage.

## Highlights

• Both stunning methods and storage time affected antioxidant status of fish.
• The decrease of antioxidant capacity led to oxidation of myofibrillar protein.
• Ice/water slurry and gill cut damaged gelation properties of myofibrillar protein.
• A schematic illustration of myofibrillar protein oxidations was proposed.
• Rapid stunning methods have great potential in the aquatic processing industry.

## Introduction

Myofibrillar proteins (MPs) accounts for 55 ∼ 60% of the total protein in fish muscles, and it is a structural protein with important biological functions. MPs are closely related to the water retention, elasticity, and texture of meat products. Protein oxidation has become one of the focuses of food research in recent years (Li et al., 2020). In fish and fish products, reactive oxygen species (ROS) can directly catalyze muscle protein oxidation (Yu, Xu, Jiang, & Xia, 2017), especially MPs, which are susceptible to ROS. In addition, secondary products of lipid oxidation can also lead to the oxidation of MPs (Luna & Estévez, 2019). The oxidation of MPs leads to the loss of sulfhydryl (Sun, Kong, Liu, Zheng, & Zhang, 2021), the increase of carbonyl content (Cheng et al., 2022), and the polymerization of proteins. Therefore, the MPs oxidative could lead to the destruction of their biochemistry and structure, which finally reduce the nutritional values of fish and fish products (Estévez & Xiong, 2019).

Stress is a non-specific response of animals in the face of endogenous or exogenous stress (Zhang, Wang, Dong, Mei, & Xie, 2023), which is one of the causes of physiological hemostatic dysfunction and deterioration of fish quality (Bermejo-Poza et al., 2021). The stunning methods have a certain influence on the biochemical process of postmortem muscle. Stress increases muscle activity and ATP degradation, thus resulting in detrimental effects on the texture, color perception, and shelf life of fish fillets (Bondoc, 2002, Bondoc, 2014). Some studies have shown that coma/slaughter antemortem and postmortem stress accelerates the formation of ROS, resulting in oxidative modification of lipids and proteins (Xing, Gao, Tume, Zhou, & Xu, 2018). Percussion and stabbing are the two main stunning methods widely used in the Chinese fishery industry. The extreme stress caused by fish stunning has deleterious effects on the postmortem structural integrity of MPs, possibly resulting in changes in MPs oxidation and functional properties (Zhao et al., 2022).

MPs are an essential protein in gel formation. Therefore, the gelation process of MPs is related to the changes in structural and functional properties of MPs (Li, Wang, Kong, Shi, & Xia, 2019), among which myosin is the most closely related. Gel properties directly determine the quality of surimi products. Good MPs gels exhibit ideal texture and WHC based on compact network structure (Xia, Kong, Xiong, & Ren, 2010), but the gelation of MP becomes worse with the deepening of oxidation (Zhang et al., 2019). However, the stunning procedure changes the structural integrity of MPs, which leads to decreased gelation properties. Large yellow croaker, *Larimichthys crocea*, is distributed on the southeast coast of China. At present, large yellow croaker is a marine fish with delicious taste, high nutritional value and great market potential that is considered to be one of the most commercially and economically valuable fish in China (Ma, Mei, & Xie, 2021). However, the effects of stunning methods on the MPs and gelation properties have not been reported. Therefore, the objective of the present study was to analyze the effects of stunning methods on physiochemical, antioxidant process, and gelation properties of MPs from large yellow croaker.

## Material and methods

### Sample preparation

**Fig. 1:**
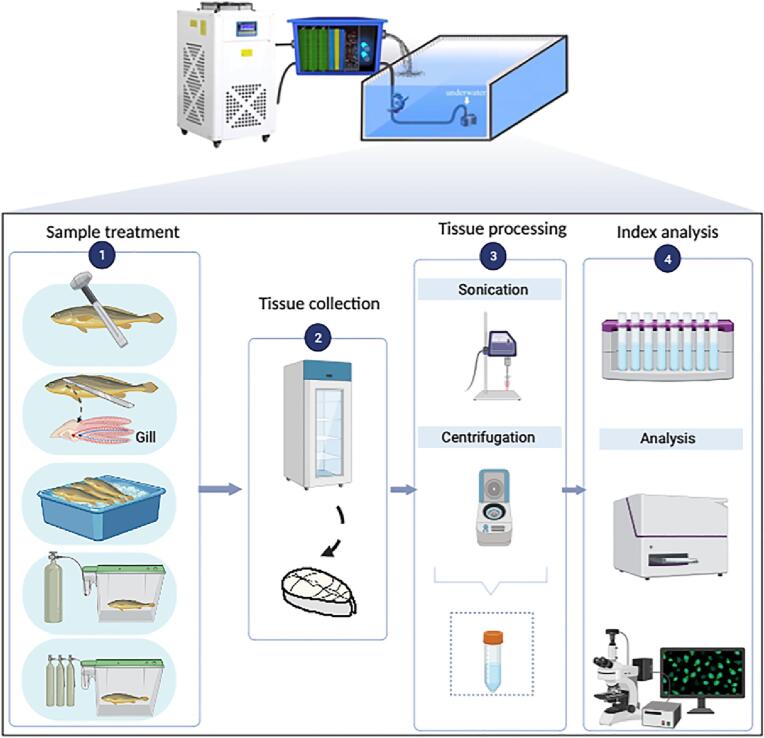
Schematic diagram showing the experimental design.

Live large yellow croaker (body length of 30 ± 5 cm; body weight 500 ± 20 g) were obtained from a local seafood market in Lingang New Town (Shanghai, China) and transported to the laboratory by live fish truck. After arriving in the laboratory, fish were rested in 500 L water tanks with dissolved oxygen for 24 h to restore the pressure that may arise during transportation. Large yellow croakers (n = 105) with the same body size and respiratory rate were selected and randomly divided into five groups and then stunned by trained lab members in the slaughtered lab (Fig. 1):

T_1_: hit on the head (n = 21) with a stick.

T_2_: fish (n = 21) were cut the gill blood vessels and returned to the water (20 °C, the dissolved oxygen was 6–8 mg/L) for exsanguination.

T_3_: fish (n = 21) were immersed in a bucket with a 1:1 ratio of ice to water (3 ± 1 °C).

T_4_: fish (n = 21) were asphyxiated with food-grade CO_2_ (20 °C).

T_5_: fish (n = 21) were asphyxiated with 40% CO_2_ + 30 % N_2_ + 30% O_2_.

The stunning standards for fish were loss of balance, cessation of eye movements (breathing), and no response to external stimuli. Afterward, the stunned large yellow croaker was slaughtered and rinsed with deionized water. Then the samples were packaged separately in polyethylene bags and stored in the refrigerator at 4 °C. The back flesh was taken randomly to measure at 0, 12th, 24th, 60th, 96th, 132th, and 168th h, respectively.

### Extraction of MPs

MPs were extracted using the description of Pei, Mei, Wu, Yu, and Xie (2022). The weighed 2 g of fish meat was mixed with 20 mL of Tris-Maleate (0.05 mol/L KCl) and centrifuged at 10,614 × g (15 min, 4 °C). The residue was mixed with 20 mL of Tris-Maleate (0.05 mol/L, pH 7.0) and processed following the above method. The sediment was mixed with 20 mL Tri-Maleate (0.6 mol/L KCl, pH 7.0) evenly after standing at 4 °C for 3 h. The supernatant obtained by centrifugation according to the above parameters was MPs.

### Carbonyl contents

The carbonyl contents of the MP solution were measured according to the approach of Bian, Yu, Yang, Mei, and Xie (2022). Ten millimeters of 2,4- dinitrophenyl hydrazine (DNPH) was mixed with 1 mL MPs solution at 25 °C for 1 h. The mixture was precipitated with 1 mL 20% trichloro- acetic acid (TCA) solution and centrifuged at 2,500 × *g* for 15 min at 4 °C. The supernatant was discarded and the precipitate was mixed with 1 mL ethanol: ethyl acetate (1:1, v/v) containing 10 mM HCl. Then the resulting pellet was incubated after dissolving in 6 M guanidine hydrochloride at 37 °C for 16 min. The absorbance was then measured at 370 nm and the free carbonyl compounds content was expressed as μmol/g protein.

### Ca^2+^-ATPase activity

2 g flesh were added with 18 mL normal saline and homogenized in ice water bath. The mixture was centrifuged at 2,500 × *g* for 15 min at 4 °C. The supernatant (10% of the homogenate supernatant) was diluted 10 times to 1% with normal saline. The activity of Ca^2+^-ATPase in actomyosin was determined by micro method, and the kit was purchased from Nanjing Jiancheng Bioengineering Institute, China. The absorbance was read at 636 nm. Finally, the results were presented as U mgprot^−1^.

### Free amine content

The OPA solution (40 mg OPA, 1 mL methanol, 1.095 g borax, 20% SDS, 100 μL β-mercaptoethanol) was diluted to 50 mL with distilled water. OPA solution should be protected from light. 4 mL OPA solution was fully mixed with 200 μL MPs (3 mg/mL) at 35 °C for 2 min. A fluorescence spectrophotometer (F-7100) was used to determine the absorbance of the solution at 340 nm. The free amino content was calculated by the standard curve of l-leucine.

### Dityrosine content

The dityrosine content of MPs was determined by the approach of Zheng, Zhou, Zhang, Wang, and Wang (2021). MP was diluted to 1 mg/mL in phosphate buffer (20 mM, pH 6). A fluorescence spectrophotometer (F-7100) was used to determine the absorbance of the solution (Ex = 325 nm, Em = 420 nm).

### MPs solubility

The MPs solubility was determined as described by Li et al. (2019). Protein content in MPs was determined by the biuret method. 10 mL MPs samples were centrifuged at 10,000 × *g* for 20 min at 4 °C. The MPs solubility was expressed in the samples as follows:

$$MPs\;solubility=\frac{MPs\;content\;after\;centrifugation}{MPs\;content\;before\;centrifugation}\times 100\%$$

### Muscle antioxidant status

The activities of total superoxide dismutase (SOD), catalase (CAT), and glutathione peroxidase (GPx) were determined by commercial kits purchased from Nanjing Jiancheng Research Institute (China).

2 g flesh were added with 18 mL normal saline and homogenized in ice water bath. The mixture was centrifuged at 2,500 × *g* for 15 min at 4 °C. The supernatant was used to determine SOD, CAT and GPx.

The SOD activity was carried out following the guidance for the commercial kit (Nanjing Jiancheng Research Institute, China). The sample of 20 uL was mixed with WST-1 working solution (160 uL). 20 uL reaction initiating solution was added to the mixture solution and placed it at 37 °C for 30 min. The absorbance was read at 450 nm.

Double distilled water was placed in 1 cm light diameter quartz cuvette to zero adjustment. The pre-treated sample 0.02 mL was added to the bottom of the cuvette. The substrate solution (3 mL) was quickly added to the cuvette. The absorbance was read at 240 nm (A_1_). The absorbance was measured again immediately in 1 min (A_2_).

$$CAT\;(U∙mg^{-1}\;prot)=764.5\times (A_{1}-A_{2})÷Protein\;concent$$

GPx activity was expressed by the oxidation rate of GSH and the decrease of GSH per unit time. The reaction of GSH with 5.5-Dithiobis-2-nitrobenzoic acid (DTNB) can produce yellow 5-thio-2-nitrobenzoic acid anion catalyzed by GPx. The product had a maximum absorption peak at 423 nm. Finally, the decrease of GSH caused by non-enzymatic reaction must be deducted when calculating the activity of this enzyme.

### Preparation of MPs gels

Surimi was suspended in ice water. The mixture was centrifuged at 3,000 × g for 15 min at 4 °C to achieve the purpose of dehydration. The sediment was mixed with 50 mM PIPES (NaCl, pH 6.0). The mixture was heated at 35 °C for 60 min and then transferred to 90 °C (30 min) for further heating. After heating, the sample is immediately placed in the refrigerator (4 °C) to cool overnight.

### Whiteness and WHC of MPs gel

The color of the gel was measured by a colorimeter (CR-400, Konica Minolta, Tokyo, Japan). Whiteness was expressed as follows:

$$\mathrm{Whiteness}=100-\sqrt{{\left(100-L^{*}\right)}^{2}\;+a^{*2}+b^{*2}}$$

The gels (5 g) were centrifuged at 5,000 × g for 15 min at 4 °C. WHC (%) was expressed as follows:

$$WHC\;\left(\%\right)=\left(1-\frac{W_{0}-W_{1}}{W_{0}}\right)\times 100\%$$

### Water mobility and distribution of MPs gel

An LF-NMR (Niumag MesoMR23-060H. I, Suzhou, China) analyzer was used to measure the gel water mobility and distribution. The gel was cut into pieces (20 × 20 × 20 mm) and placed in NMR tubes. Corresponding to the pulse sequence of Carr-Purcell- MeiboomGill, 21 MHz was selected as the frequency of proton resonance. 3000 echoes and 16 scans were included in each measurement. The pseudo color images of proton density-weighted of samples were obtained by performing magnetic resonance imaging (MRI).

### Confocal laser scanning microscopy (CLSM)

The gel microstructure was observed using a 20x water (Numerical Aperture) immersion objective lens. The gel samples were freeze-sectioned, and slice thickness was 10 μm. The obtained samples were frozen at −20 °C until use. The gel slices were stained with rhodamine B (0.025 g/ml) for 10 min, and the excess dyes were washed off. The fluorescence intensity was recorded from 568 to 590 nm.

### Statistical analysis

All analyses were repeated in triplicate, and the results were expressed as mean ± standard deviation (SD). Two-way analysis of variance (ANOVA) was used for multiple comparisons using SPSS 22.0 software. Duncan's multi-range test was used to determine the difference between averages, and chart making was performed using Origin software.

## Results and discussion

### Carbonyl contents

**Fig. 2:**
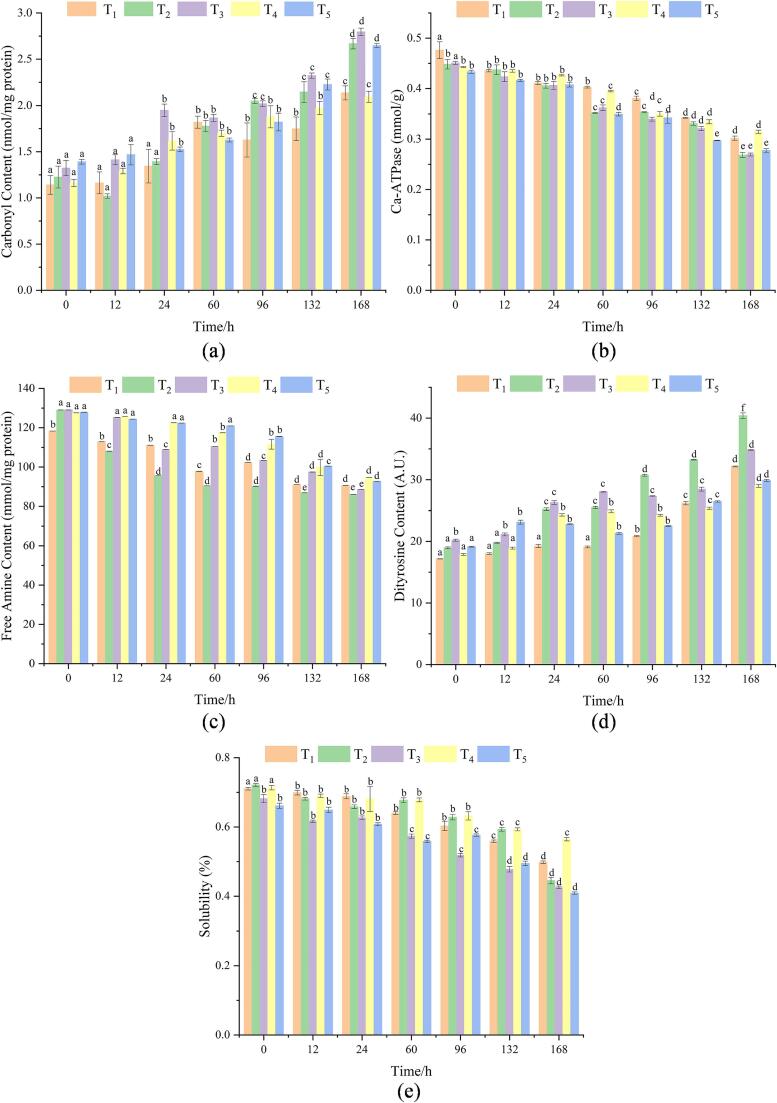
Changes in carbonyl content (a), Ca^2+^-ATPase (b), free amine content (c) and dityrosine (d), and protein solubility (e) of fillets treated with different stunning methods during chilled storage (4 °C). Different lowercase letters indicate that there are significant differences among groups (*P* < 0.05).

Carbonyl contents are used to assess the degree of oxidative denaturation of proteins (Zhang, Fang, Hao, & Zhang, 2018). Carbonyl contents can evaluate the degree of oxidative modification of MPs by ROS (Zhang, Bian, et al., 2023). The changes in carbonyl contents under different stunning methods are shown in Fig. 2a. There was no significant difference in carbonyl contents among different treated samples at 0 h (*P* > 0.05). Carbonyl contents increased the increase of storage time, and the carbonyl contents of T_2_, T_3_, and T_5_ at 168 h were 2.4 times, 2.12 times, and 1.90 times that at 0 h, respectively. Zhang, Li, Hong, and Luo (2021) indicating that stressful gill cut stunning facilitated the carbonylation process of MPs in silver carp fillets, which was similar to the current study. The decrease in antioxidant enzyme activity led to the oxidation of MPs during cold storage (Zhang, Li, Jia, Huang, & Luo, 2018). The carbonyl contents of T_2_, T_3_, and T_5_ samples at 168 h were significantly higher than that of T_1_ and T_4_ samples (*P* < 0.05), indicating that both stunning stress and storage time promoted in terms of elevated carbonyl content. External stimulation could cause excessive accumulation of ROS in muscle by destroying mitochondrial function and inhibiting ATP synthesis (He et al., 2019). Therefore, low temperature or hypoxia inhibited ROS elimination and electron transport, which led to high oxidation sensitivity of MPs (Lushchak, 2011). Zhang et al. (2019) also pointed out that the gill cut samples contained higher carbonyl contents, which was similar to the current study.

### Ca^2+^-ATPase activities

Ca^2+^-ATPase activity has been widely used to measure actomyosin integrity and monitor changes in fish during storage (Shui et al., 2022). The Ca^2+^-ATPase activities in all samples decreased while the Ca^2+^-ATPase activities in T_2_ and T_3_ samples decreased at the fastest rate during cold storage, reaching 59.82 and 59.78 % on the 168th h, respectively (Fig. 2b). This is in accordance with results of carbonyl contents, suggesting that T_2_ and T_3_ fillets were more prone to be attacked by ROS and had higher susceptibility to oxidative modifications. The activities of Ca^2+^-ATPase in all samples decreased, indicating the fish produced a complex series of reactions with the extension of storage time. In general, cold preservation inhibits the Ca^2+^-ATPase activities (Mahato, Zhu, & Sun, 2023), which is also supported by our result. The contents of Ca^2+^-ATPase in T_2_, T_3_, and T_5_ samples were significantly higher than in T_1_ and T_4_ samples at 168 h of storage (*P* < 0.05). This may be due to the large number of free radicals generated by the high oxidative stress of T_2_, T_3_, and T_5_ samples, leading to the oxidation of sensitive proteins, such as Ca^2+^- ATPase. Shao et al. (2018) reported that the decrease in Ca^2+^-ATPase activities was attributed to protein denaturation. Zhang et al. (2021) reported the effect of percussion and gill cutting on the structural changes and oxidation state of MPs in silver carp. They found the stunning stress increased the susceptibility of fish to oxidative modification.

### Free amine content

Protein oxidation initiated by free radicals leads to oxidative modification of side chain amino acids. Free amino content is one of the most important indicators of protein oxidative damage. The free amine content of all samples showed a downward trend. The free amino content of T_2_ samples decreased by 33.3% during cold storage, which was significantly faster than other samples (*P* < 0.05) (Fig. 2c). At the end of storage, the T_2_ samples had the lowest free amine content (86.09 ± 0.04 mmol/mg protein). According to Zhang et al. (2017) study, compared with non-stressful percussive stunning, gill cut stunning accelerated anaerobic metabolic process (e.g., depletion of glycogen, accumulation of lactic acids, and degradation of adenosine triphosphates) of silver carp, which induced obvious reduction in muscular pH, thereby facilitating protein oxidative damage. No significant difference was observed in free amine contents among T_1_, T_4_, and T_5_ samples (*P* > 0.05). The carbonyl groups formed by the oxidation of ε-NH_2_ lysine residue could react with the amino group, leading to a decrease in free amine content (Cao & Xiong, 2015). In addition, the addition of 1% SDS during the determination destroyed the non-covalent interaction between proteins, indicating that the ε-NH_2_ loss may be caused by the covalent addition of oxidized hydroquinone and non-protein amines. Li et al. (2020) reported that the loss of free amine was also affected by free radicals. Similarly, Zhang, Li et al. (2018) showed that the stress caused by the stunning methods could reduce the antioxidant capacity of fish, causing MPs to be easily oxidized by hydroxyl radicals.

### Dityrosine content

During food storage, two l-tyrosine molecules are coupled through protein oxidation to form covalent bonds to produce dityrosine, which promotes protein crosslinking. Therefore, the content of dityrosine can be used to evaluate the degree of protein cross-linking and amino acid changes (Zhang, Fang, Hao, & Zhang, 2018). As shown in Fig. 2d, the content of dityrosine in the T_3_ sample was significantly higher than that in other samples (*P* < 0.05). T_3_ samples had the highest dityrosine content at 0 h, which could be related to environmental factors, and low temperature resulted in the increased oxidative stress of fish. However, the content of dityrosine in all samples also increased significantly (*P* < 0.05) with the increase of storage time, which was caused by the MPs oxidation during storage. Colombo et al. (2015) indicated that the increase in the dityrosine content of protein was caused by the deepening of protein oxidation. At the end of storage, T_2_ samples had significantly higher dityrosine content than the other samples (*P* < 0.05), and the dityrosine content of T_4_ samples was the lowest. It could be ascribed that the cutting of the gills caused the stress response of the fish, which produced free radicals and promoted the oxidation of tyrosine.

### MPs solubility

Solubility is an important functional property that can reflect the degree of protein denaturation and aggregation (Du et al., 2021). The MPs solubility of T_3_ and T_5_ samples was significantly lower than that of T_1_, T_2_, and T_4_ at 0 h (*P* < 0.05) (Fig. 2e). The MPs solubility of each sample decreased significantly with increasing storage time (*P* < 0.05), however, the decrease in the T_4_ sample was not obvious. Li et al. (2020) confirmed that the decrease in MPs solubility was related to the increase in oxidative denaturation and cross-linking of proteins. Zhang et al. (2021) pointed that gill cut-stunned fillets exhibited more severe loss of tryptophan fluorescence both in no oxidized or highly oxidized MPs, indicating the oxidative denaturation of the protein occurs. The MPs solubility of T_4_ samples was significantly higher than that of other samples at the end of storage (*P* < 0.05). Stunning stress accelerated the glycolysis rate of fish and promoted the subsequent production of lactic acid, resulting in faster ATP consumption and a decrease in pH value. The changes in pH value changed the microenvironment around MP and made MPs close to the isoelectric point, resulting in a decrease in the solubility of MPs (Li, Zheng, Xu, Zhu, & Zhou, 2018). Addis et al. (2012) also found that the high-stress stunning method caused serious MP oxidative damage.

### Antioxidant enzymes

CAT, SOD, and GPx can chelate toxic superoxide radicals and H_2_O_2_ to protect biologically from oxidative damage (Ma, Wang, Chen, Yu, & Han, 2021). SOD converts superoxide radicals (O^2–^) to H_2_O_2_ and O_2_, CAT decomposes H_2_O_2_ into H_2_O, and GPx detoxifies H_2_O_2_ and organic hydroperoxides to H_2_O with GSH (Cecerska-Heryć et al., 2021). The antioxidant enzyme activities of all samples increased at the beginning and then decreased (Table 1). The antioxidant enzymes could remove ROS and maintain the dynamic balance of the antioxidant system in the early stage of cold storage. The decrease of antioxidant enzyme activities in the later stage could be due to the denaturation of intracellular proteolytic enzymes. At the end of storage, impaired CAT activities were observed in all samples. Similarly, Yu et al. (2017) also reported the CAT activities of grass carp fillets decreased sharply during cold storage. The initial CAT activities in T_1_, T_4_, and T_5_ samples were significantly higher than that of the T_2_ and T_3_ samples (*P* < 0.05). However, Zhang, Li, et al. (2018) found that ice/water slurry and gill cut samples had higher initial CAT activity, which was not consistent with our results. Differences in fish species and low-temperature duration may lead to differences in results (Fadhlaoui & Couture, 2016). The CAT activities of T_2_ and T_3_ samples were significantly lower than that of other samples in the later stage of storage (*P* < 0.05). The decreased CAT activity could in turn induce postmortem oxidation in fish. The GPx and SOD activities in T_2_ and T_3_ samples were significantly higher than those of T_1_, T_4_, and T_5_ samples at 0 h (*P* < 0.05), indicating that low temperature and asphyxia may affect the GPx and SOD activities (Lushchak, 2011). In T_2_ and T_3_ samples, the stress generated by stunning was responsible for the faster occurrence of peak GPx and SOD activities. It can be seen that the fish fillets with ice/water slurry, gill cut, and long storage time are more likely to be oxidized due to the lack of antioxidant enzyme activities. The results of CAT, GPX, and SOD activities indicated that stunning stress had a great effect on the antioxidant enzymes activities of fish fillets, especially in T_2_ and T_3_ samples with the lowest antioxidant enzyme activities. Protein oxidation usually occurs in the condition of an imbalance between the scavenging capacity of antioxidants and the generation of free radicals. Combined with the results of protein oxidation levels, it could be considered that the decrease of antioxidant enzymes activity for scavenging ROS was the major reason for those undesirable chemical reactions.

**Table 1:** Changes in total superoxide dismutase (SOD), catalase (CAT), and glutathione peroxidase (GPx) activities of fillets treated with different stunning methods during chilled storage (4 °C).

	Groups	0	12	24	60	96	132	168
SOD	T_1_	8.67 ± 0.86^a^	9.17 ± 0.12^a^	10.71 ± 0.85^b^	25.09 ± 1.47^c^	17.22 ± 0.10^b^	13.24 ± 0.08^b^	7.84 ± 0.07^a^
	T_2_	7.44 ± 0.06^a^	8.81 ± 0.21^a^	10.60 ± 0.09^b^	13.41 ± 0.50^b^	8.41 ± 0.19^a^	7.41 ± 0.25^a^	2.88 ± 0.16^a^
	T_3_	8.26 ± 0.47^a^	17.73 ± 0.52^b^	19.17 ± 0.73^b^	18.21 ± 0.20^b^	17.83 ± 0.25^b^	8.26 ± 0.47^a^	7.69 ± 0.09^a^
	T_4_	6.41 ± 0.63^a^	10.28 ± 0.70^b^	12.86 ± 0.40^b^	32.57 ± 0.24^d^	21.83 ± 0.28^c^	15.59 ± 0.52^b^	9.66 ± 0.16^a^
	T_5_	7.85 ± 0.65^a^	8.90 ± 0.23^a^	10.47 ± 0.13^b^	11.63 ± 0.26^b^	6.88 ± 0.42^a^	6.39 ± 0.50^a^	6.09 ± 0.44^a^

CAT	T_1_	6.07 ± 0.47^b^	8.17 ± 0.40^a^	8.01 ± 1.00^a^	9.33 ± 0.38^a^	6.33 ± 0.95^b^	4.84 ± 0.63^c^	1.87 ± 0.46^e^
	T_2_	5.89 ± 0.61^c^	7.03 ± 1.19^b^	7.54 ± 0.11^b^	6.73 ± 0.45^b^	7.23 ± 0.27^b^	1.70 ± 0.39^e^	0.93 ± 0.12^e^
	T_3_	5.48 ± 0.73^c^	7.62 ± 0.23^b^	8.31 ± 0.14^a^	8.68 ± 0.29^a^	5.85 ± 0.65^c^	2.06 ± 0.38^d^	0.99 ± 0.06^e^
	T_4_	6.85 ± 0.54^b^	8.93 ± 0.38^a^	9.63 ± 0.49^a^	9.15 ± 0.61^a^	5.83 ± 0.45^c^	5.18 ± 0.22^c^	2.81 ± 0.48^d^
	T_5_	6.85 ± 0.57^b^	6.08 ± 0.39^b^	7.35 ± 0.40^b^	5.09 ± 0.10^c^	4.59 ± 0.11^c^	3.14 ± 0.45^d^	1.40 ± 0.52^e^

GPx	T_1_	30.34 ± 0.05^b^	35.44 ± 1.08^b^	36.12 ± 0.89^b^	25.88 ± 0.07^c^	22.42 ± 0.18^c^	18.95 ± 0.07^d^	17.55 ± 0.53^d^
	T_2_	45.09 ± 2.04^a^	41.21 ± 0.44^a^	27.00 ± 0.14^c^	23.72 ± 0.99^c^	20.01 ± 0.87^c^	15.46 ± 0.41^d^	7.64 ± 1.32^e^
	T_3_	36.50 ± 1.31^b^	41.92 ± 0.95^a^	28.09 ± 1.44^c^	26.18 ± 0.03^c^	23.87 ± 0.85^c^	16.13 ± 0.93^d^	10.97 ± 1.05^d^
	T_4_	31.06 ± 0.86^b^	36.49 ± 1.56^b^	42.44 ± 1.17^a^	42.48 ± 0.33^a^	28.79 ± 0.95^c^	22.85 ± 1.14^c^	21.12 ± 1.21^c^
	T_5_	36.84 ± 0.17^b^	37.04 ± 0.16^b^	37.55 ± 0.07^b^	35.55 ± 0.05^c^	24.72 ± 1.28^c^	18.38 ± 0.67^d^	13.39 ± 1.26^d^

Note: a. Results are presented as mean ± standard deviations. Different letters indicated significant difference (*P* < 0.05). b. T_1_: hit on the head; T_2_: gill cut; T_3_: immersion in ice/water slurry; T_4_: CO_2_ narcosis; T_5_: 40% CO_2_ + 30% N_2_ + 30% O_2_.

### Whiteness of MPs gel

**Fig. 3:**
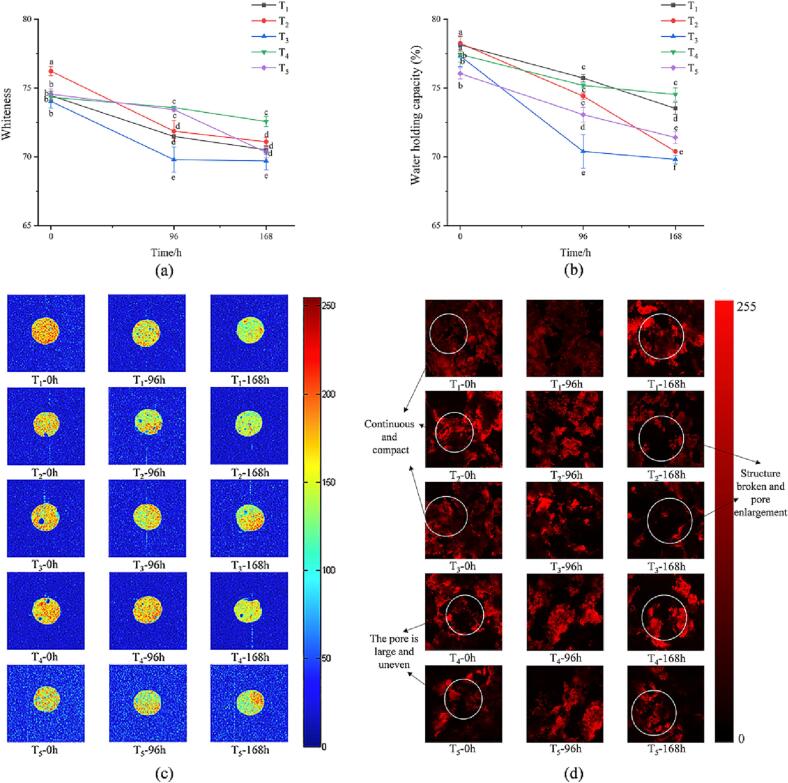
Changes in whiteness (a), water hold capacity (b), proton density image (c), and CLSM microstructures (d) of myofibrillar protein gels of large yellow croaker stunned by different methods. Different lowercase letters indicate that there are significant differences among groups (*P* < 0.05). (For interpretation of the references to color in this figure legend, the reader is referred to the web version of this article.)

The whiteness is affected by the degree of protein denaturation or water-holding capacity of gel. The gel of the T_2_ sample had the highest whiteness value at 0 h (Fig. 3a) as the lighter muscle color was caused by bloodletting. Van Nguyen (2018) confirmed that bloodletting reduced Hb, Mb, and white blood cells in fish meat, thus delaying the oxidation of lipids and heme proteins in fish meat. The gel whiteness of all samples decreased with the extension of storage time. Some studies have found that the oxidative denaturation of MPs led to the reduction of free water within the gel, which affected the whiteness of the gel (Cao et al., 2022). The gel whiteness of T_4_ samples was significantly higher than that of other samples at the end of storage (*P* < 0.05). The decrease in gel whiteness of other samples may be due to a weakened antioxidant defense system, which affects sensitivity to MPs oxidation and postmortem oxidation. In the current study, the lower whiteness values observed in T_3_ samples may be due to an attempt to escape causing the blood to leave the blood vessels and concentrate in the muscle, resulting in the increased red intensity of surimi (de Melo et al., 2021). Xia et al. (2010) suggested that the decrease in gel whiteness was related to a non-enzymatic Browning reaction, which occurred between the amines in proteins and lipid oxidation products. The decrease in gel whiteness in this study may be due to shock stress-induced excess production of free radicals, leading to protein and lipid peroxidation damage (Li et al., 2020).

### WHC of MPs gel

WHC reflects the water retention ability of the MPs gel, which is an important parameter of fish products (Li et al., 2019). The higher the WHC, the more water is combined or retained in the MPs gel network. As shown in Fig. 3b, the WHC of all samples decreased as the formation of loose MPs gel during cold storage. The oxidation of MPs weakens the ability of the gel network structure to retain water (Cheng et al., 2022), consistent with our results. The MPs gel WHC of the T_2_ sample was the highest compared with other samples at the beginning. Zhang et al. (2019) indicated that slight oxidation could enhance the cohesion of the MPs gel and lead to a compact gel structure. Therefore, the initial gel WHC of T_2_ samples was kept at a high level. The MPs gel WHC of T_2_ and T_3_ samples were significantly lower than that of other samples at 168 h (*P* < 0.05). The accelerated decomposition of proteins in fish fillets destroys the integrity of myosin, resulting in the destruction and weakening of the reticular structure of the gel (Xiong, Blanchard, Ooizumi, & Ma, 2010). Therefore, severe oxidative modifications decreased WHC in MP gels, especially in ice/water and gill cut samples at 168 h. Addis et al. (2012) found that the high-stress slaughter method could destroy the integrity of myosin. At the same time, the modification of amino acid residues in MPs led to the destruction of hydrogen bond interaction between gel and water, resulting in gel water loss (Utrera & Estevez, 2012).

### Water mobility and distribution of MPs gel

The water distribution and relaxation time of MPs gel obtained under different stunning methods were analyzed by LF-NMR. The enhanced mobility of water indicates that it is loosely bound to macromolecules, resulting in a longer relaxation time of water. The relaxation time of all samples increased with storage time, indicating increased mobility of water. Table 2 exhibits a similar change in relaxation time between T_2b1_ and T_2b2_. In the beginning, the relaxation time of T_2b1_ and T_2b2_ in T_2_ and T_3_ samples was significantly higher than that of other samples (*P < 0.05*), which may be due to the loose binding of bound water to protein macromolecules.

**Table 2:** Changes in water distribution of myofibrillar protein gels of large yellow croaker stunned by different methods at storage.

Water dynamics	Stunning methods	Storage time (h)		
		0	96	168
T_2b1_	T_1_	0.28 ± 0.12^e^	0.59 ± 0.14^d^	0.68 ± 0.16^c^
	T_2_	0.45 ± 0.05^d^	0.47 ± 0.07^d^	0.41 ± 0.12^d^
	T_3_	0.43 ± 0.00^d^	0.50 ± 0.02^d^	1.02 ± 0.03^a^
	T_4_	0.37 ± 0.04^e^	0.28 ± 0.12^e^	0.89 ± 0.13^b^
	T_5_	0.34 ± 0.03^e^	0.64 ± 0.22^c^	0.83 ± 0.20^b^

T_2b2_	T_1_	1.77 ± 0.68^e^	5.07 ± 1.23^a^	4.08 ± 0.60^b^
	T_2_	3.53 ± 0.17^c^	4.06 ± 0.20^b^	3.96 ± 0.77^c^
	T_3_	4.51 ± 0.44^b^	3.80 ± 0.56^c^	4.40 ± 0.28^b^
	T_4_	2.90 ± 2.27^d^	3.42 ± 0.33^c^	5.00 ± 0.25^a^
	T_5_	3.18 ± 0.00^c^	4.35 ± 0.21^b^	4.58 ± 0.11^b^

T_21_	T_1_	95.48 ± 0.00^e^	121.81 ± 5.98^b^	114.19 ± 16.76^c^
	T_2_	106.02 ± 5.20^d^	95.48 ± 0.00^e^	130.57 ± 6.41^a^
	T_3_	121.81 ± 5.98^b^	121.69 ± 6.14^b^	117.87 ± 11.55^c^
	T_4_	102.59 ± 10.06^d^	113.64 ± 5.58^c^	102.59 ± 10.06^d^
	T_5_	109.70 ± 0.00^d^	121.81 ± 5.98^b^	121.81 ± 5.98^b^

T_22_	T_1_	1011.64 ± 0.00^f^	1296.89 ± 190.31^c^	1335.45 ± 0.00^d^
	T_2_	1290.67 ± 63.33^d^	1123.34 ± 55.12^e^	1534.37 ± 0.00^a^
	T_3_	1245.88 ± 0.00^d^	1173.42 ± 125.94^e^	1290.67 ± 63.33^d^
	T_4_	1209.91 ± 177.54^d^	1053.06 ± 154.53^f^	1053.06 ± 154.53^f^
	T_5_	1245.88 ± 0.00^d^	1290.67 ± 63.33^d^	1123.34 ± 55.12^e^

Note: a. Results are presented as mean ± standard deviations. Different letters indicated significant difference (*P* < 0.05). b. T_1_: hit on the head; T_2_: gill cut; T_3_: immersion in ice/water slurry; T_4_: CO_2_ narcosis; T_5_: 40% CO_2_ + 30% N_2_ + 30% O_2_.

The increase of T_2b1_ and T_2b2_ during storage was the result of the unfolding of the MPs structure, resulting in a decrease in the WHC of MPs (Sun et al., 2021). The main moisture in MPs gel is immobile water. On 0 day, the T_21_ relaxation time of T_3_ samples was significantly longer than that of other samples (*P* < 0.05), which was associated with the MPs denaturation caused by low-temperature stress. The T_21_ of all samples continued to increase during storage. At 168 h, the T_21_ relaxation time of the T_2_ samples was the longest followed by T_5_, T_3_, T_1_, and T_4_ samples. The increase of T_21_ may be due to the unfolded MPs structure, exposing more hydrophobic groups, and reducing the solubility of proteins, which affected the formation of the MPs gel structure. In addition, the T_21_ of T_2_ samples was significantly higher than that of other samples. MPs were oxidized to form larger MPs aggregates, thereby forming a loose gel network and reducing the WHC of the gel (Zheng et al., 2021). The changes of T_22_ were similar to those of T_21_ in all samples during cold storage. In addition, the T_22_ of the gill cut stunning was longer than that of the other samples at 168 h, indicating a change in water-protein interactions. The increase in T_22_ indicated an increase in the possibility of water migration to free water. Therefore, the storage time and stunning methods could affect the water mobility of fish fillets. In conclusion, MPs gel from gill cut samples exhibited higher immobilized water losses at the end of storage.

The proton density image of the MPs gel samples is shown in Fig. 3c. Magnetic resonance imaging (MRI) can describe the internal structure state and proton distribution of gel. The red area of the MPs gel in each sample decreased after cold storage. However, T_2_ samples were almost no red region after cold storage, which was consistent with the results of WHC. In addition, the red area of T_4_ samples was evenly distributed. Consistent with the relaxation time of WHC and T_22_, stunning methods affected the functional and structural properties of MPs, thereby reducing the MPs gel formation and hydration properties.

### Confocal laser scanning microscopy (CLSM)

The microstructure of MP gel mainly refers to the gel network structure, which is closely related to protein aggregation, intermolecular interaction, chemical force, and so on (Liao et al., 2022). As shown in Fig. 3d, the protein structure of T_1_, T_2_, and T_3_ samples was continuous and the pore size was uniform compared with T_4_ samples, which could be due to the slight oxidation caused by stunning-stress resulting in a more orderly gel network structure. Li and Xiong (2015) indicated that proper oxidation led to the different degrees of unfolding and cross-linking of MPs through hydrophobic interactions and disulfide bonds, which contributed to the formation of the gel structure. The MPs gel structure of all samples broke and the pore size became larger with the extension of storage time. The reticular structure of T_2_ and T_3_ samples became worse at the end of storage compared with other samples. A recent study reported that a dense and uniform network structure was beneficial for the gel to capture excess water (Du et al., 2022), which supported the results of WHC and LF-NMR. Therefore, severe oxidative stress promoted the fracture of the MPs gel network, resulting in the decrease of hardness and cohesion of MPs gel.

### Schematic illustration

**Fig. 4:**
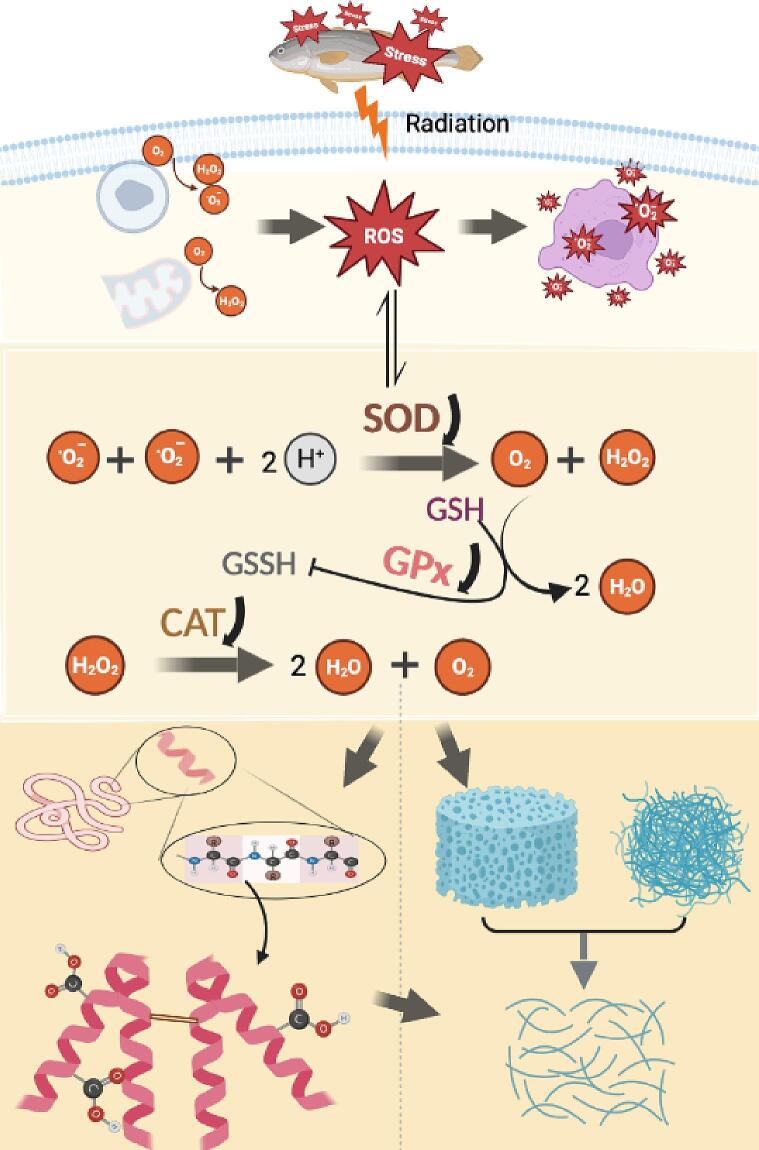
Proposed mechanism on stunning method-induced MPs oxidation. ROS: reactive oxygen species; SOD: superoxide dismutase; GPx: glutathione peroxidase; CAT: catalase.

The stunning methods promoted the oxidation level of MPs in large yellow croaker by the production of free radicals and the destruction of the antioxidant enzyme system (Fig. 4). Firstly, the activities of peroxides (SOD, CAT, GPx) changed in different degrees by different stunning methods, and the contents of carbonyl and free amines increased significantly, suggesting that the destruction of the antioxidant enzyme system could lead to MPs oxidation. In addition, the increase of MPs oxidation sensitivity leads to further aggregation and denaturation of MPs. Finally, the destruction of the antioxidant enzyme system and the strong oxidative modification of MPs led to a destroyed gel network structure and a change in water distribution.

## Conclusion

The present study indicated that stunning stress affected the antioxidant status, MPs functional properties, MPs gel properties, and water distribution of large yellow croaker after slaughter. Stunning stress in T_2_ and T_3_ samples resulted in damage to CAT, GPx, and SOD activities and increased sensitivity to postmortem oxidation. T_2_ and T_3_ resulted in the generation of MPs carbonyl, the decrease of Ca^2+^-ATPase, free ammonia and MPs solubility, and the production of dityrosine in large yellow croaker. The MPs gel properties of the gill cutting and the ice water samples reduced greatly, along with the higher immobilized water migration and free water released. In addition, cold storage time was the key factor affecting the MPs gel properties and the reticular structure of MPs gel destroyed at the end of cold storage. T_4_ samples maintained good antioxidant enzyme activities, MP structure, and gel properties during cold storage, followed by T_1_ and T_5_. In the process of stunning, the traditional gill cut and ice water should be avoided to obtain a better-quality fish product. Therefore, rapid stunning methods have great potential in the aquatic processing industry.

## Declaration of Competing Interest

The authors declare that they have no known competing financial interests or personal relationships that could have appeared to influence the work reported in this paper.

## Data Availability

Data will be made available on request.
